# Supplementary material for: Models of care for Indigenous children in rural and remote settings: A global scoping review
Source: PLOS Glob Public Health. 2025 Jul 17;5(7):e0004934. doi: 10.1371/journal.pgph.0004934 (PMC12270103; doi:10.1371/journal.pgph.0004934)
Supplement: S1 Text — (DOCX) [file pgph.0004934.s001.docx]

**Appendix 1: Complete Search Strategies**

**MEDLINE**

ex ’education’ OR exp ‘ownership’ OR exp ‘patient reported outcome measures’ OR exp ‘quality improvement’ OR exp ‘health services administration’ OR exp ‘quality of life’ OR exp ‘health care facilities, manpower, and services' OR exp ‘patient satisfaction’ OR exp ‘community mental health services’ OR exp ‘mental health services’ OR exp ‘criminology’ OR exp ‘patient care’ OR ‘holistic health’ OR exp ‘evidence-based practice’ OR exp ‘policy’ OR exp ‘health planning’ OR ‘model of care’ OR ‘care coordination’ OR ‘wraparound’ OR ‘first 2'000 days’ OR ‘outreach’ OR ‘patient reported’ OR ‘patient reported experience measures’ OR ‘delivery of healthcare’ OR ‘health services’ OR ‘health care economics’ or exp ‘health care economics and organizations’ OR ‘Referral’ OR ‘collaborative care’ OR ‘patient centered care’ OR ‘integrated care’ OR ‘multidisciplinary care’ OR ‘intersectoral care’ OR ‘care pathway’ OR ‘continuous quality improvement

AND

Exp ‘child’ OR exp ‘child, preschool’ OR exp pediatrics OR exp ‘adolescent’

AND

Exp ‘rural health’ OR exp ‘rural population’ OR exp ‘rural, hospitals’, OR exp ‘rural nursing’, OR exp ‘rural ‘’ or exp ‘remote consultation’ OR ‘rural’ OR ‘remote’ OR ‘outback’

AND

Exp ‘Indigenous Canadians’ OR exp ‘health services, indigenous’ OR exp ‘Indigenous peoples’ OR exp ‘oceanic ancestry group’ OR exp ‘Indians, North America’ or ‘First Nation’ OR ‘Indigenous’ OR ‘Aboriginal’ OR ‘australoid’

limited to

yr = 1990 until May 30, 2024

language = English

**EMBASE**

exp Education/ or exp "ownership and management"/ or exp "patient-reported outcome"/ or exp "total quality management"/ or exp patient satisfaction/ or (model of care or care coordination or wraparound or "First 2*000 days" or outreach or "patient reported experience measures").mp. or delivery of healthcare.mp. or exp health service/ or exp "quality of life"/ or health services.mp. or exp "health care management"/ or exp Community Mental Health Service/ or exp mental Health Service/ or exp Criminology/ or exp Patient Care/ or "integrative medicine"/ or exp Evidence based Practice/ or exp health care policy/ or exp health care planning/ or (Referral or "collaborative care" or "patient?centered care" or "integrated care" or "multi?disciplinary care" or "intersectoral care" or "care pathway" or "continuous quality improvement").mp.

AND

Child/ or infant/ or newborn/ or Pediatrics/ or Adolescent/

AND

exp "rural health nursing"/ or exp "rural hospital"/ or exp Rural Health/ or exp rural health care/ or Rural Population/ or exp teleconsultation/ or remote.mp. or outback.mp.

AND

exp Indigenous people/ or Indigenous.mp. or exp Indigenous health care/ or American Indian/ or exp First Nation/ or Oceanic Ancestry Group/ or Aborigin*.mp. or Australoid.mp.

limited to

yr = 1990 until May 30, 2024

language = English

**Web of Science**

education OR ownership OR 'patient reported outcome measure' OR 'quality improvement' OR 'patient satisfaction' OR 'model of care' OR 'first 2000 days' OR outreach OR 'patient reported experience measures' OR 'delivery of healthcare' OR 'health services administration' OR 'quality of life' OR 'health services' OR 'community mental health services' OR 'mental health service*' OR 'crimin*' OR 'patient care' OR 'holistic health' OR 'evidence based practice' OR 'health policy' OR 'health planning'

AND

child OR infant OR neonate OR pediatric* OR paediatric* OR adolescent

AND

'rural health' OR 'rural population' OR 'rural hospital*' OR 'rural nursing' OR 'rural health service' OR 'teleconsultation' OR 'remote'

AND

'Indigenous' OR 'American Indians' OR 'Aborigin*' OR 'oceanic ancestry group'

**SCOPUS**

TITLE-ABS-KEY(

(education OR ownership OR "patient reported outcome measure*" OR "quality improvement"

OR "patient satisfaction" OR "model of care" OR "first 2000 days" OR outreach

OR "patient reported experience measure*" OR "delivery of healthcare"

OR "health services administration" OR "quality of life" OR "health services"

OR "community mental health service*" OR "mental health service*"

OR crimin* OR "patient care" OR "holistic health" OR "evidence based practice"

OR "health policy" OR "health planning")

AND

(child OR infant OR neonate OR pediatric* OR paediatric* OR adolescent)

AND

("rural health" OR "rural population" OR "rural hospital*" OR "rural nursing"

OR "rural health service*" OR teleconsultation OR remote)

AND

(Indigenous OR "American Indian*" OR Aborigin* OR "oceanic ancestry group")

)

AND PUBYEAR > 1990 (until May 30, 2024)

AND LANGUAGE(English)

**CINAHL**

( (MH "Education" OR MH "Ownership" OR "patient reported outcome measure*" OR "quality improvement"

OR "patient satisfaction" OR "model of care" OR "first 2000 days" OR outreach

OR "patient reported experience measure*" OR "delivery of healthcare"

OR MH "Health Services Administration" OR "quality of life" OR "health services"

OR MH "Community Mental Health Services" OR MH "Mental Health Services"

OR crimin* OR "patient care" OR "holistic health" OR MH "Evidence-Based Practice"

OR MH "Health Policy" OR MH "Health Planning")

AND

(child OR infant OR neonate OR pediatric* OR paediatric* OR adolescent)

AND

("rural health" OR "rural population" OR "rural hospital*" OR "rural nursing"

OR "rural health service*" OR "teleconsultation" OR remote)

AND

(Indigenous OR "American Indian*" OR Aborigin* OR "oceanic ancestry group") )

LIMITS: Published from 1990 (till May 2024), English Language
