# Supplementary material for: Models of care for Indigenous children in rural and remote settings: A global scoping review
Source: PLOS Glob Public Health. 2025 Jul 17;5(7):e0004934. doi: 10.1371/journal.pgph.0004934 (PMC12270103; doi:10.1371/journal.pgph.0004934)
Supplement: Attachment — Submitted filename: Response to Reviewers.docx [file pgph.0004934.s004.docx]

# Editor and reviewer feedback

We thank the editor and reviewers for their time working on this paper with us. Please see our responses in red text below.

| **Date:** | Feb 21 2025 05:08PM |
| --- | --- |
| **To:** | "Joseph Freeman" jfre7317@uni.sydney.edu.au |
| **cc:** | kalarania@newgen.co |
| **From:** | "PLOS Global Public Health" globalpubhealth@plos.org |
| **Subject:** | PLOS Global Public Health Decision: Revision required [PGPH-D-24-02491] |

PGPH-D-24-02491
Models of care for Indigenous children in rural and remote settings: a global scoping review
PLOS Global Public Health

Dear Dr. Freeman,

Thank you for submitting your manuscript to PLOS Global Public Health. After careful consideration, we feel that it has merit but does not fully meet PLOS Global Public Health’s publication criteria as it currently stands. Therefore, we invite you to submit a revised version of the manuscript that addresses the points raised during the review process.

==============================

Academic Editor Guillaume Fontaine, PhD: Thank you for your submission. In addition to the reviewers' comments, please address the following points in your revision:

1. Introduction: Please remove the sub-headings in this section, these are not necessary. Please review to ensure that each paragraph of the introduction is well defined, and flows well to the next one. Sentences like "the paper chooses the term..." are usually not seen in the introduction and belong to the methods when defining concepts.

Sub-headings removed with thanks. Also, reviewed to ensure flow, and moved the latter mentioned phrase to methods per your advice.

2. The PRISMA-ScR is a reporting checklist, and not methodological standards. Were you also guided by scoping review methodological standards (e.g., JBI, Arksey and O'Malley).

Thank you for this very good point. The JBI’s updated guidance on scoping reviews from 2021 was used but was not mentioned in the paper as it is aligned and consistent with the PRISMA-ScR. Both are now named and cited.

3. Please attached all search strategies used as appendices.

Thank you for the suggestion – complete search strategies now attached as an appendix.

4. The analysis section is very short, and it is unclear exactly what was done. Please expand this section and give concrete examples.

Analysis section significantly revised with thanks.

5. The section "How outcomes were measured" is insufficiently detailed. It would be relevant to include descriptive statistics on the frequency of different outcome measures, more extensive descriptions of these measures, and examples.

The analysis section has been revised and expanded to include proportions of types, summaries of each qualitative paper. Thank you for the helpful request.
==============================

Entire manuscript revised for minor corrections. Thank you for your time reviewing this work.

=================================

Please submit your revised manuscript by Apr 07 2025 11:59PM. If you will need more time than this to complete your revisions, please reply to this message or contact the journal office at globalpubhealth@plos.org. When you're ready to submit your revision, log on to https://www.editorialmanager.com/pgph/ and select the 'Submissions Needing Revision' folder to locate your manuscript file.

Please include the following items when submitting your revised manuscript:

- A rebuttal letter that responds to each point raised by the editor and reviewer(s). You should upload this letter as a separate file labeled 'Response to Reviewers'.
- A marked-up copy of your manuscript that highlights changes made to the original version. You should upload this as a separate file labeled 'Revised Manuscript with Track Changes'.
- An unmarked version of your revised paper without tracked changes. You should upload this as a separate file labeled 'Manuscript'.

Guidelines for resubmitting your figure files are available below the reviewer comments at the end of this letter.

We look forward to receiving your revised manuscript.

Kind regards,

Guillaume Fontaine, PhD, RN
Academic Editor
PLOS Global Public Health

Journal Requirements:

Additional Editor Comments (if provided):

N/A


[Note: HTML markup is below. Please do not edit.]

Reviewers' comments:

Reviewer's Responses to Questions

**Comments to the Author**

1. Does this manuscript meet PLOS Global Public Health’s [publication criteria](https://journals.plos.org/globalpublichealth/s/criteria-for-publication)? Is the manuscript technically sound, and do the data support the conclusions? The manuscript must describe methodologically and ethically rigorous research with conclusions that are appropriately drawn based on the data presented.

Reviewer #1: Partly

Reviewer #2: Yes

Reviewer #3: Yes

Reviewer #4: Partly

2. Has the statistical analysis been performed appropriately and rigorously?

Reviewer #1: N/A

Reviewer #2: Yes

Reviewer #3: Yes

Reviewer #4: N/A

3. Have the authors made all data underlying the findings in their manuscript fully available (please refer to the Data Availability Statement at the start of the manuscript PDF file)?

The [PLOS Data policy](https://journals.plos.org/globalpublichealth/s/data-availability) requires authors to make all data underlying the findings described in their manuscript fully available without restriction, with rare exception. The data should be provided as part of the manuscript or its supporting information, or deposited to a public repository. For example, in addition to summary statistics, the data points behind means, medians and variance measures should be available. If there are restrictions on publicly sharing data—e.g. participant privacy or use of data from a third party—those must be specified.

Reviewer #1: Yes

Reviewer #2: Yes

Reviewer #3: Yes

Reviewer #4: Yes

4. Is the manuscript presented in an intelligible fashion and written in standard English?

PLOS Global Public Health does not copyedit accepted manuscripts, so the language in submitted articles must be clear, correct, and unambiguous. Any typographical or grammatical errors should be corrected at revision, so please note any specific errors here.

Reviewer #1: Yes

Reviewer #2: Yes

Reviewer #3: Yes

Reviewer #4: Yes

5. Review Comments to the Author

Please use the space provided to explain your answers to the questions above. You may also include additional comments for the author, including concerns about dual publication, research ethics, or publication ethics. (Please upload your review as an attachment if it exceeds 20,000 characters)

Reviewer #1: The article addresses a critical and under-researched topic. While it has merit, the methodological section requires improvement. The identified methodological limitations raise concerns about potential biases and the external validity of the findings. Furthermore, the argumentation could benefit from better sequencing of cause-effect links and more robust referencing to support key claims.

We have addressed this through substantial expansion and revision of the methodology section particularly, including the analysis section, and made changes throughout the manuscript in response to this and other similar comments from reviewers. Thank you for your comment.

Reviewer #2: *the paper acknowledges Indigenous perspectives on health but it does not center them in the study's framework. It would be beneficial to explicitly incorporate an Indigenous-led research paradigm to reinforce self-determination.

Thank you for this comment. We recognize the significance of Indigenous-led research paradigms in reinforcing self-determination. Our study aims to incorporate Indigenous perspectives on health, including by evaluating all included studies using the well-regarded Aboriginal and Torres Strait Islander Quality Appraisal Tool. The study was informed by multiple Indigenous health paradigms as outlined in the introduction. Multiple Indigenous co-authors - including the senior author - helped to centre our research framework on Indigenous principles. However, this study is a review which follows the appropriate JBI methodological guidance and this is acknowledged in the limitations section of our manuscript. We have ensured that our discussion reflects the ongoing structural challenges and have added brief clarifications where relevant.

*conclusion could be strengthened by advocating for co-designed, Indigenous-led models of care rather than externally imposed ones

This last paragraph has been revised to include the above statement with thanks.

*the paper acknowledges colonisation and systemic racism, however the discussion would benefit from a stronger critique of how colonial healthcare structures continue to shape Indigenous child health outcomes.

Thank you for your comment. This idea has now been significantly expanded upon in the limitations section and the paragraph preceding the limitations section.

*The paper recommends realist evaluation for future reviews. While useful, this is still a Western evaluation framework, next time consider advocating for Indigenous evaluation frameworks.

Thank you for the feedback. We recommended realist evaluation as this emerged from the existing literature - however, even more importantly we recommend using Indigenous evaluation frameworks. We have now amended the manuscript including the conclusion to reflect this, and cited examples of Indigenous evaluation frameworks.

This is an important and well-structured paper that amplifies the need for Indigenous-led, culturally secure healthcare models for Indigenous children in remote areas. By strengthening its methodological grounding in Indigenous research paradigms, reinforcing Indigenous leadership, and deepening the discussion of systemic challenges, this paper has the potential to shift the discourse from deficit-based models to strength-based Indigenous solutions.

Thank you for your comments

Reviewer #3: Well done to the authors.

While you spent some time talking about how different entities frame and operationalize model of care, your manuscript would benefit from an articulation of what a model of care entails exactly, at least from your own perspective after consulting all of these sources.

Thank you for this suggestion. The 4^th^ last paragraph in Discussion section is now dedicated to this.

Also, you might want to pay attention to your formatting and keep it consistent. In some cases, you start with 'six' of 16 studies... and then continue with ‘4’【1,22 m】 of 16 studies in the same sentence. You should choose to either spell out the numbers and keep that consistent or use their digit.

Thank you for your feedback. Relevant sentences now re-written to avoid first word being a number, obviating issue (priorly written following convention of spelling numbers if first word in sentence).

Reviewer #4: Title: Models of care for Indigenous children in rural and remote settings: a global scoping review
ϖ Finding and assessing successful care models for Indigenous children in rural and isolated places is the goal of this assessment. This research aims to inform the creation of contextually relevant and culturally suitable healthcare models that cater to the special requirements of Indigenous children by drawing on experiences from throughout the world.
ϖ The use of the pronoun (we) is not encouraged, so please rephrase the sentence.

Thank you for recommendation. The pronoun ‘We’ has been removed throughout the manuscript except for in the section ‘Acknowledgement of Country’ which was written by our Australian Aboriginal co-author and in which we feel it is appropriate to retain his more personal chosen words.

ϖ participants were less than or equal to 18 years of age (child is used to refer to this age group): Needs more clarifications as childhood (3 to 11 years old), adolescence, or teenage (from 12 to 18 years old)?

Revised to the following for clarity: “study participants were less than or equal to 18 years of age (this study uses the term ‘child’ for those aged 0-18 years)”

ϖ In the text, when referring to the author et al., please include the No. of references following the author et al.

Implemented throughout with thanks

ϖ Where is the statistical analysis?

Thank you for this feedback which was mirrored in the Editors’ comments above. The statistical section has been revised (please see manuscript and reply to Editor’s comments).

ϖ Please include a few lines explaining the conclusion of this study.

Conclusion now revised addressing above with thanks.

ϖ Grammar: Needs minor corrections throughout the manuscript.

The entire manuscript has been thoroughly revised for minor corrections. Thank you for your time reviewing this work.

6. PLOS authors have the option to publish the peer review history of their article ([what does this mean?](https://journals.plos.org/globalpublichealth/s/editorial-and-peer-review-process#loc-peer-review-history)). If published, this will include your full peer review and any attached files.

If agreeable to reviewers we accept publication of peer review history.

**Do you want your identity to be public for this peer review?** If you choose “no”, your identity will remain anonymous but your review may still be made public.


For information about this choice, including consent withdrawal, please see our [Privacy Policy](https://www.plos.org/privacy-policy).

Reviewer #1: No

Reviewer #2: No

Reviewer #3: No

Reviewer #4: **Yes:** Sura Saad Abdullah

[NOTE: If reviewer comments were submitted as an attachment file, they will be attached to this email and accessible via the submission site. Please log into your account, locate the manuscript record, and check for the action link "View Attachments". If this link does not appear, there are no attachment files.]

While revising your submission, please upload your figure files to the Preflight Analysis and Conversion Engine (PACE) digital diagnostic tool, https://pacev2.apexcovantage.com/. PACE helps ensure that figures meet PLOS requirements. To use PACE, you must first register as a user. Registration is free. Then, login and navigate to the UPLOAD tab, where you will find detailed instructions on how to use the tool. If you encounter any issues or have any questions when using PACE, please email PLOS at figures@plos.org. Please note that Supporting Information files do not need this step.

Figures have been produced and reviewed on PACE and appear of publishable quality.
