# Supplementary material for: Models of care for Indigenous children in rural and remote settings: A global scoping review
Source: PLOS Glob Public Health. 2025 Jul 17;5(7):e0004934. doi: 10.1371/journal.pgph.0004934 (PMC12270103; doi:10.1371/journal.pgph.0004934)
Supplement: Attachment — Submitted filename: Response_to_Reviewers_auresp_2.docx [file pgph.0004934.s005.docx]

# Editor feedback

We thank the Editor for inviting this minor revision. Please see our responses in red text below.

PGPH-D-24-02491
Models of care for Indigenous children in rural and remote settings: a global scoping review
PLOS Global Public Health

1. The methods section of the abstract is underdeveloped; consider cutting back the background and conclusion of the abstract to add to the methods what methodological guidelines were followed, eligibility criteria, study selection and data extraction process and data analysis methods.

Methods section significantly expanded and other areas of abstract made more succinct – overall abstract word count is unchanged.

2. Sources: You conducted searches in different bibliographical databases. Were other sources searched, considering this is a scoping review? For example, grey literature sources (Google Scholar, Open Grey), government reports, dissertations and theses, hand-searching of relevant journals? If these were not included as sources, why?

Thank you for your comment. We acknowledge that the inclusion of grey literature is an important aspect of comprehensive scoping reviews. In this review, we focused our search on peer-reviewed literature through multiple bibliographical databases to ensure the quality and relevance of sources. This is a limitation of our study, and it is now addressed in the discussion to guide future research efforts.

New section for discussion section:

*This review did not include grey literature such as government reports or unpublished studies, which may have excluded relevant insights, particularly from community-based or policy sources. This could be addressed in future research for a more comprehensive understanding.*

3. Sources: The sentence "High-level MeSH headings were used to encompass keywords (e.g., holistic, systems, models, models of care etc.). The MEDLINE strategy is shown in Box 1 and was adapted to all other databases. The complete search strategies are provided in Appendix 1." goes into search strategy.

Thank you for this observation. Section moved as recommended.
